# Financial illiteracy among internal medicine, surgery, and radiology residents regarding medical imaging costs in the Netherlands

**DOI:** 10.1007/s00330-025-11510-7

**Published:** 2025-03-20

**Authors:** Ton Velleman, Rudi A. J. O. Dierckx, Yfke P. Ongena, Klaas P. Koopmans, Walter Noordzij, Thomas C. Kwee

**Affiliations:** 1 Departments of Radiology, Nuclear Medicine and Molecular Imaging, Medical Imaging Center, University Medical Center Groningen, University of Groningen, Groningen, The Netherlands; 2 Faculty of Arts, Communication and Information Sciences, University of Groningen, Groningen, The Netherlands

**Keywords:** Medical imaging, Financial management, Residency, Education

## Abstract

**Purpose:**

To assess the knowledge of internal medicine, surgery, and radiology residents of medical imaging costs at a university hospital in the Netherlands.

**Methods:**

A survey was conducted among internal medicine, surgery, and radiology residents at a tertiary care university hospital to determine their knowledge and view on medical imaging costs. Participants were asked to estimate the costs of a two-view chest X-ray, unenhanced CT of the brain, unenhanced MRI of the brain, contrast-enhanced CT of the chest and abdomen, ultrasound of the complete abdomen, and FDG-PET and PSMA-PET torso. Estimates within ± 25% of the available published costs were considered accurate.

**Results:**

A total of 44 participants (18 in internal medicine, 15 in surgery, and 11 in radiology) were included. No resident accurately estimated all imaging costs, with accuracies ranging from 18% for contrast-enhanced CT of the chest and abdomen to 39% for two-view chest X-rays. Cost estimation accuracy did not significantly vary by specialty or training duration. Most participants were concerned about the affordability of medical care within or beyond the next five years (80%, 95%), 66% of residents felt that doctors bear responsibility for limiting healthcare costs, and 89% agreed that education about the financial aspects of medical imaging is useful.

**Conclusion:**

This study showed that residents are financially illiterate regarding medical imaging costs, and neither the duration of training nor specialty influences their knowledge levels. Nevertheless, residents share common concerns and responsibilities about rising healthcare costs and express a desire for additional education regarding the finance of medical imaging.

**Key Points:**

***Question*** *Assessing the knowledge levels of residents regarding medical imaging costs provides valuable information for policymakers involved in the design of medical curricula*.

***Findings*** *Residents from internal medicine, surgery, and radiology demonstrate limited knowledge of medical imaging costs but appear eager to learn*.

***Clinical relevance*** *There is a need to educate residents about the costs of medical imaging, promote the efficient use of limited resources, and reduce overall healthcare expenses*.

**Graphical Abstract:**

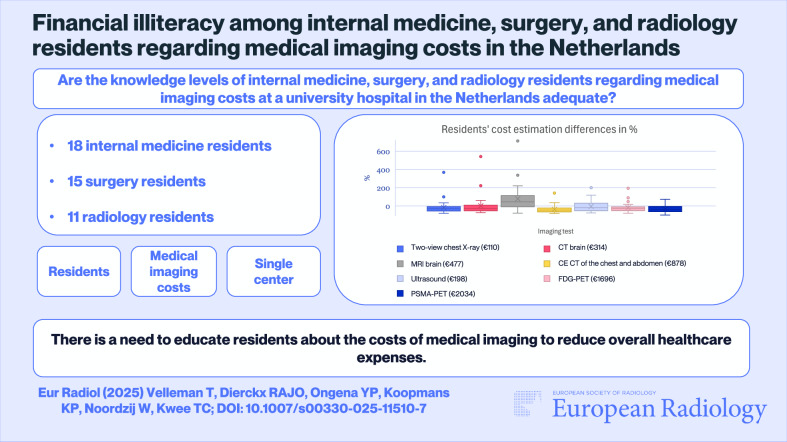

## Introduction

Rising healthcare costs pose a significant challenge to maintaining the affordability and accessibility of healthcare in the Western world. However, financial education within the medical curriculum for physicians in training appears to be insufficiently addressed [1–4]. Medical imaging constitutes a substantial and continuously growing portion of healthcare expenditures [5]. The demand for medical imaging has surged exponentially over the past decades and is projected to continue to grow in the foreseeable future [6, 7].

Radiologists, as key advisors to clinicians, must be knowledgeable in order to recommend the most appropriate and cost-effective imaging tests. Internal medicine physicians and surgeons, being frequent users of these imaging services, should also possess a fundamental understanding of imaging costs. This could potentially reduce unnecessary and costly testing and mitigate the risk of overdiagnosis—defined as a process where the risk of harm of an intervention outweighs its benefits [8, 9].

However, it remains unclear whether residents in internal medicine, surgery, and radiology are adequately informed about the costs associated with medical imaging and what their attitudes are toward healthcare costs related to medical imaging. Previous studies investigating residents’ knowledge of medical imaging costs have primarily been conducted in the United States, with a notable lack of studies in Europe. Should financial literacy be found lacking, this may prompt policymakers to consider integrating this topic into residency curricula.

Therefore, the purpose of this study was to assess the knowledge levels of internal medicine, surgery, and radiology residents regarding the costs of medical imaging at a university hospital in the Netherlands.

## Materials and methods

### Medical Ethics Review Board

Approval from the local medical ethics review board was obtained for this prospective survey (IRB number: 19198).

### Settings and participants

Residents and doctors-not-in-training from the internal medicine and surgery departments of a tertiary care university hospital in the Netherlands (serving a population of over 2.5 million people) were invited to participate in an online questionnaire on medical imaging costs during a group meeting held between April 4th and May 18th, 2024. They were not informed about the contents of the meeting in advance and completed the questionnaire without access to online or other resources, under the supervision of one of the authors (T.V.). Radiology residents were approached individually to participate and completed the questionnaire without access to online or other resources, under the supervision of one of the authors (T.V.).

Residents from departments other than internal medicine, surgery, and radiology were excluded from the analysis to maintain group homogeneity. No other exclusion criteria were applied.

The questionnaire was accessed via a QR code provided during the meeting, which participants scanned using their smartphones, after which the questionnaire became available for completion on their devices. Participation was voluntary, and informed consent was automatically obtained upon participation. Registration was conducted via IP addresses to ensure unique participants were included.

### Questionnaire

The questionnaire was developed in Qualtrics by the authors, including two radiologists, a nuclear medicine physician, and a survey specialist. To ensure the quality of reporting, the “Checklist For Reporting Results of Internet E-surveys” has been used as a reporting standard guideline specific to survey results. The questionnaire comprised a total of thirteen questions: twelve closed-ended questions, one open-ended question, and two conditional open-ended questions.

The questionnaire collected data on the following variables: residents’ age, gender, department (internal medicine, surgery, radiology, or other), years of residency training, and prior experience with medical imaging costs (an optional open-ended question was included to allow for further clarification). Participants were also asked how frequently they requested various imaging examinations (plain radiograph, ultrasound, CT, MRI, and FDG-PET), with response options ranging from “never” to very often”.

Additionally, the questionnaire included six Likert-type statement items with five levels of agreement (ranging from “strongly disagree” to “strongly agree”). These statements addressed concerns about the affordability of medical care within and/or after the next five years, whether residents consider costs when requesting medical imaging, the perceived responsibility of doctors to limit healthcare costs, the consideration of cost-saving possibilities when requesting medical imaging (e.g., preventing other unnecessary costly tests or reducing/preventing morbidity and mortality with appropriate imaging), and the usefulness of financial education on imaging costs prior to residency. Responses of “agree” or “strongly agree” were grouped to determine the highest level of agreement among residents.

Participants were also asked to estimate the costs of several of the most frequently requested imaging tests at our institution, with options ranging from €0 to €5000. The imaging tests included a two-view chest X-ray (€110), unenhanced CT of the brain (€314), unenhanced MRI of the brain (€477), contrast-enhanced CT of the chest and abdomen (€878), ultrasound of the complete abdomen (€198), FDG-PET (€1695), and PSMA PET torso (€2034). The used imaging costs are based on mutual medical service costs in the Netherlands, which are accessible through the hospital’s intranet [10]. Note that the costs for FDG PET and PSMA PET did not include a diagnostic CT. To exclude any question-order effects, the above-mentioned diagnostic tests were presented in random order. Supplementary File A contains the complete list of questions and answer options.

### Data analysis

Residents’ survey responses, including demographic data (age, gender, years of residency training, department) were collected and analyzed. The duration of residency training was categorized into four groups for analytical purposes; 1. Not in training, 2. Short training experience (< 1–3 years), 3. Medium training experience (4–7 years), and 4. Long training experience (7 years or more), which could result from part-time work schedules, pregnancy, illness, or other factors extending the training beyond the typical 5–6 years.

The residents’ estimated costs for the seven different medical imaging tests were extracted from the published costs available in the Netherlands, resulting in ‘cost estimation differences’ (in Euros). For example, the cost of an unenhanced CT of the brain (€314) minus a resident’s estimate of €250 results in a cost estimation difference of €64. This allowed us to calculate error percentages and identify residents whose estimates were within ± 25% of the published cost for each of the seven imaging tests, consistent with previous research on cost estimation accuracy [11–13]. Estimates outside of this ± 25% range were considered incorrect. The proportion of residents whose estimates fell within ± 25% of the published costs was calculated for the entire group and per department (internal medicine, surgery, and radiology).

A multiple linear regression analysis was conducted to explore associations between the independent variables (duration of residency training and department) and the dependent variable (cost estimation difference). *p*-values of less than 0.05 were considered statistically significant.

All data were analyzed using IBM SPSS Statistics for Windows, version 23.

## Results

### Residents’ characteristics

From the initial group of participants, approximately 30 internal medicine residents, 20 surgery residents, and 16 radiology residents were in training in their respective departments during the survey period. Out of these, 46 residents completed the questionnaire. Two residents employed in other departments were excluded from the analysis, leaving 44 completed questionnaires for further evaluation, resulting in a response rate of 67%. Of the included residents (18 in internal medicine, 15 in surgery, and 11 in radiology), 21 were male, and 23 were female, with a mean age of 32 years (range: 25–38 years). All questionnaires were fully completed.

Among the respondents, 35 were currently in training, while 9 were doctors not in training. The average duration of residency training was 4.6 years (range: 0–9 years). Four residents (9%) reported having prior knowledge of medical imaging costs, which they had acquired through previous studies, internships, scientific research, projects related to the finance of medical imaging, or personal interest.

The imaging modalities most frequently requested by residents were conventional X-ray studies and CT scans (requested by 33 residents, 75%) and ultrasound imaging (requested by 28 residents, 64%) (Table 1).

**Table 1:** Frequency of the different imaging modalities that are requested by the 44 residents

Modality	Often^*^	Sometimes	Never
X-ray	33 (75%)	6 (14%)	5 (11%)^**^
Ultrasound	28 (64%)	12 (27%)	4 (9%)
CT	33 (75%)	7 (16%)	4 (9%)
MRI	16 (36%)	22 (50%)	6 (14%)
PET	18 (41%)	18 (41%)	8 (18%)

^*^ “Often” is defined as an examination that is requested weekly or even daily, “sometimes” is defined as an examination that is requested only sporadically

^**^ Among the five residents who have never requested plain radiographs; four are radiology residents

### Cost estimation error and associated variables

**Fig. 1:**
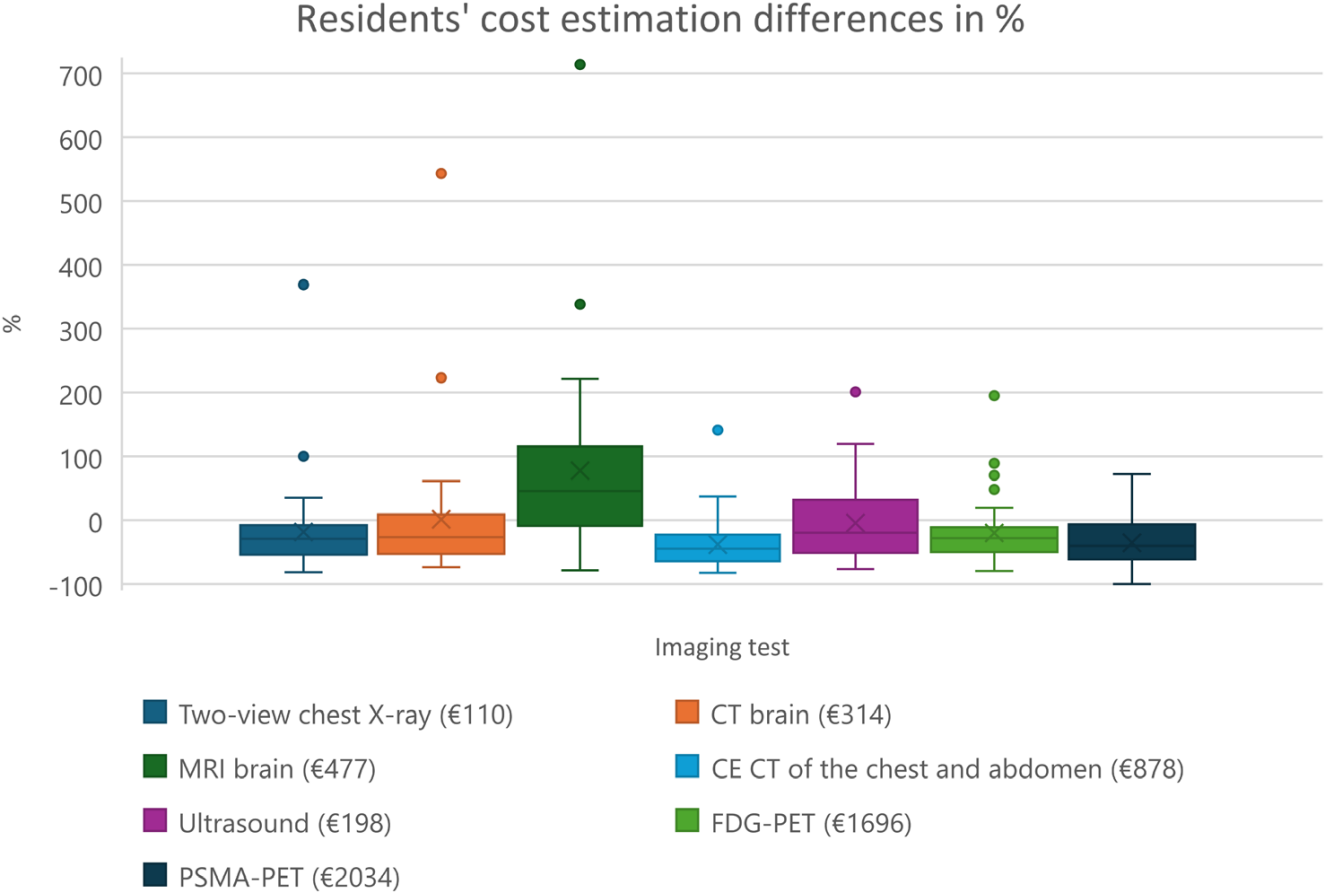
Residents’ cost estimation differences are in % relative to the published costs** of several imaging tests. * Shown are the actual prices per modality and their corresponding resident cost estimation differences in ±%. ** Publicly available cost in the Netherlands [10]

The percentage of residents who estimated costs within ± 25% of the published costs for the seven medical imaging tests ranged between 18% for contrast-enhanced CT of the chest and abdomen to 39% for two-view chest X-rays. Table 2 and Fig. 1 provide detailed values and their distributions.

**Table 2:** The cost estimation differences (the difference between the published examination cost and the resident’s estimates) in Euros

Imaging test	Published cost^**^	Mean estimate	SD	Median estimate	Min–Max (€)	Within ± 25% of the published costs (%)
Two-view chest X-ray	€ 110,-	€ 89,-	76	€ 78,-	20–516	39%
Unenhanced CT of the brain	€ 314,-	€ 318,-	331	€ 230,-	83–2020	32%
Unenhanced MRI of the brain	€ 477,-	€ 846,-	669	€ 692,-	101–3883	27%
Contrast-enhanced CT of the chest and abdomen	€ 878,-	€ 541,-	356	€ 490,-	152–2120	18%
Ultrasound of the complete abdomen	€ 198,-	€ 189,-	120	€ 159,-	45–595	27%
FDG-PET torso*	€ 1695,-	€ 1351,-	829	€ 1209,-	335–5000	27%
PSMA (prostate-specific membrane antigen)-PET torso	€ 2034,-	€ 1309,-	716	€ 1211,-	0–3505	23%

^*^ FDG- PET and PSMA PET scans without diagnostic CT

** Publicly available cost in the Netherlands [10]

For internal medicine residents, the percentage of accurate cost estimations within ±25% of the published cost for the seven medical imaging tests ranged from 17% for unenhanced MRI of the brain, contrast-enhanced CT of the chest and abdomen, and ultrasound of the complete abdomen, to 33% for two-view chest X-ray, unenhanced CT of the brain, and FDG-PET torso. Surgery residents demonstrated accuracies ranging from 27% for unenhanced MRI of the brain and contrast-enhanced CT of the chest and abdomen, to 53% for two-view chest X-ray and ultrasound of the complete abdomen. Among radiology residents, the accuracies ranged from 9% for contrast-enhanced CT of the chest and abdomen and ultrasound of the complete abdomen, to 46% for unenhanced MRI of the brain (Table 3).

**Table 3:** Number of residents with cost estimates within ± 25% of the published cost per examination and department

Imaging test	Internal medicine	Radiology	Surgery
Two-view chest X-ray	6 (33%)	2 (18%)	8 (53%)
Unenhanced CT of the brain	6 (33%)	2 (18%)	6 (40%)
Unenhanced MRI of the brain	3 (17%)	5 (46%)	4 (27%)
Contrast-enhanced CT of the chest and abdomen	3 (17%)	1 (9%)	4 (27%)
Ultrasound of the complete abdomen	3 (17%)	1 (9%)	8 (53%)
FDG-PET torso^*^	6 (33%)	1 (33%)	5 (33%)
PSMA (prostate-specific membrane antigen)-PET torso	4 (22%)	2 (18%)	5 (33%)

^*^ FDG- PET and PSMA PET scans without diagnostic CT

No significant correlation was found between the cost estimation differences for the various imaging tests (two-view chest X-ray, unenhanced CT of the brain, unenhanced MRI of the brain, contrast-enhanced CT of the chest and abdomen, ultrasound of the complete abdomen, FDG PET torso, PSMA PET torso) and the duration of training or department type. Similarly, no significant correlation was identified between residents who had prior knowledge of medical imaging costs and their cost estimation accuracy.

None of the 44 residents accurately estimated the costs of all seven medical imaging tests, and 4 residents (9%) incorrectly estimated the costs of all tests.

### Residents’ attitudes towards medical imaging costs

Strong agreement among residents was observed regarding concerns about the affordability of medical care within the next five years (35 residents, 80%) and beyond five years (42 residents, 95%). Additionally, 11 residents (25%) reported considering the costs of medical imaging when requesting imaging, 29 residents (66%) felt that doctors bear responsibility for limiting healthcare costs, 17 residents (39%) considered cost-saving opportunities when requesting imaging, and 39 residents (89%) agreed that educating medical students about the financial aspects of medical imaging before residency would be beneficial.

## Discussion

This study demonstrated that residents in internal medicine, surgery, and radiology lack accurate knowledge of medical imaging costs. None of the residents accurately estimated all imaging costs (i.e., within the 25% error margin), and 9% incorrectly estimated the cost of all imaging tests. The financial (il)literacy of residents regarding medical imaging is not influenced by their years of training or specialty. Additionally, the few residents who reported prior knowledge of medical imaging finance did not perform better in estimating the costs of different modalities.

Despite their general illiteracy regarding medical imaging costs, the majority of residents expressed concerns about the affordability of healthcare both in the near and distant future. Notably, radiology residents had the least accurate estimates, although the reasons for this remain unclear. Approximately two-thirds of the participants agreed that doctors play a vital role in controlling the rise in healthcare costs, and nearly 90% of the residents expressed a desire to receive education on medical imaging costs prior to starting their residency. This suggests a clear need for financial education related to medical imaging costs during residency, or possibly even earlier in medical school, to enhance cost awareness and contribute to reducing healthcare costs through the efficient use of limited resources [14, 15].

Previous studies investigating knowledge of medical imaging costs have primarily focused on medical specialists or were conducted a considerable time ago, in the United States or South Africa, where medical costs and training curricula differ from those in Europe [12, 13, 16]. For instance, online survey studies conducted by Vijayasarathi et al in 2015 and 2016 among 381 postgraduate physician trainees and 1066 radiology trainees revealed that only 6% of the postgraduate physician trainees and 17% of the radiology trainees provided cost estimates within the correct range (defined as within ± 25% of published Medicare allowable amounts) [12, 13].

Integrating financial education early in residency training or even during medical school could potentially reduce costs by increasing knowledge, as demonstrated by previous studies involving minor educational interventions, such as lectures on abdominal imaging charges and 15-minute educational modules focused on cost-effective ordering [17]. By enhancing cost awareness among residents, they can make more informed decisions and reduce unnecessary imaging studies, as shown in a study by Kanzaria et al, where 50% of emergency physicians identified increased education as one of the most effective solutions for reducing unnecessary imaging requests [18]. Increased cost awareness regarding medical imaging through resident education may lead to more efficient use of diagnostic tests. Further research is needed to determine the most effective way to integrate financial education about medical imaging into the medical curriculum.

The study has several limitations. First, the sample size was relatively small; however, the high response rate suggests that the results accurately reflect the current level of knowledge and general opinions of internal medicine, surgery, and radiology residents in a large academic hospital. Second, only a selection of available imaging tests was presented to the participants to maintain survey conciseness, which limits the scope of the available imaging tests evaluated. Third, the questionnaire only addressed the direct costs of imaging, and not the potential long-term cost savings that imaging could provide by preventing or reducing mortality and morbidity, shortening hospital stays, and reducing the need for additional diagnostics and therapies. Fourth, this was a single-center study conducted in an academic hospital, and the results may differ in other hospitals or countries with different curricula and imaging costs. However, in the Netherlands, the training curricula are standardized nationwide, so similar results might be expected in other institutions.

In conclusion, this study demonstrated that residents are financially illiterate regarding medical imaging costs, and neither the duration of training nor the specialty appears to influence their level of knowledge. Nonetheless, residents share common concerns and responsibilities regarding rising healthcare costs and express a strong desire for additional education on the financial aspects of medical imaging.

## Supplementary information

ELECTRONIC SUPPLEMENTARY MATERIAL
