# Supplementary material for: Financial illiteracy among internal medicine, surgery, and radiology residents regarding medical imaging costs in the Netherlands
Source: Eur Radiol. 2025 Mar 20;35(9):5450–6. doi: 10.1007/s00330-025-11510-7 (PMC12350507; doi:10.1007/s00330-025-11510-7)
Supplement: Supplementary file 1 — ELECTRONIC SUPPLEMENTARY MATERIAL [file 330_2025_11510_MOESM1_ESM.pdf]

Financial illiteracy among internal medicine, surgery, and radiology residents regarding medical imaging costs in the Netherlands

ELECTRONIC SUPPLEMENTARY MATERIAL

Supplemental file A, the complete list of the questions and answer options.

Q1. What is your age?

Yes/No

Q2. What is your gender?

Male/Female/Other

Q3. Within which specialty are you working?

Internal medicine/Radiology/Surgery/Other, namely

Q4. How many years have you been in training within your specialty?

Scale from <1 year to > 10 years, or I am not in training

Q5. Have you acquired knowledge regarding the costs of radiological and/or nuclear imaging prior to beginning your specialty training?

Yes/No or I don't know

Q5a. If you answered 'yes' on the previous question, please clarify how you gained knowledge of the costs of radiological and/or nuclear imaging.

Q6. Please indicate for the following imaging tests (conventional X-ray. Ultrasound, CT, MRI, FDG-PET) what applies to you.

I request regularly/ I sometimes request/ I have never requested this imaging test

Introduction to the next questions:

Radiological and nuclear imaging (medical imaging) incurs costs. However, it can also save costs, for example, by enabling faster diagnoses that can limit or prevent morbidity and mortality, avoid unnecessary interventions, or shorten hospital stays.

The following are several statements for which you can indicate the extent to which you agree or disagree. Please rate your agreement on a five-point scale, ranging from 'strongly disagree' to 'strongly agree'.

Q7. I am concerned about the short-term affordability of healthcare over the next five years.

Q8. I am concerned about the long-term affordability of healthcare (beyond the next five years).

Q9. When requesting medical imaging, I also take the costs into consideration.

Q10. Limiting healthcare costs is key responsibility for doctors.

Q11. When requesting medical imaging, I also considering cost-saving possibilities.

Q12. It is beneficial to educate medical students about the costs of medical imaging.

Introduction to the next questions:

The following questions pertain to the costs (in euros) of the most commonly requested radiological and nuclear medicine imaging studies at our institution. Using a slider (€0 - €5000), please indicate what you believe the costs per study are. Our goal is to assess your knowledge of the current costs of medical imaging. Please provide your best estimate at this time.

Q13. What is your cost estimate for the following imaging studies at our institution: two-view chest X-ray, unenhanced CT of the brain, unenhanced MRI of the brain, contrast-enhanced CT of the chest and abdomen, ultrasound of the complete abdomen, FDG-PET, and PSMA-PET torso?

Table 1. Checklist for Reporting Results of Internet E-Surveys (CHERRIES)

	**Checklist for Reporting Results of Internet E-Surveys (CHERRIES)**		
Item Category	***Checklist Item***	***Explanation***	
**Design**			
	Describe survey design	Describe target population, sample frame. Is the sample a convenience sample? (In “open” surveys this is most likely.)	Materials & Methods Page 1-2
**IRB (Institutional Review Board) approval and informed consent process**			
	IRB approval	Mention whether the study has been approved by an IRB.	Materials & Methods Page 1
	Informed consent	Describe the informed consent process. Where were the participants told the length of time of the survey, which data were stored and where and for how long, who the investigator was, and the purpose of the study?	Materials & Methods Page 1
	Data protection	If any personal information was collected or stored, describe what mechanisms were used to protect unauthorized access.	N/A
**Development and pretesting**			
	Development and testing	State how the survey was developed, including whether the usability and technical functionality of the electronic questionnaire had been tested before fielding the questionnaire.	Materials & Methods Page 1
**Recruitment process and description of the sample having access to the questionnaire**			
	Open survey versus closed survey	An “open survey” is a survey open for each visitor of a site, while a closed survey is only open to a sample which the investigator knows (passwordprotected survey).	Materials & Methods Page 1-2
	Contact mode	Indicate whether or not the initial contact with the potential participants was made on the Internet. (Investigators may also send out questionnaires by mail and allow for Web-based data entry.)	Materials & Methods Page 1
	Advertising the survey	How/where was the survey announced or advertised? Some examples are offline media (newspapers), or online (mailing lists – If yes, which ones?) or banner ads (Where were these banner ads posted and what did they look like?). It is important to know the wording of the announcement as it will heavily influence who chooses to participate. Ideally the survey announcement should be published as an appendix.	N/A
**Survey administration**			
	Web/E-mail	State the type of e-survey (eg, one posted on a Web site, or one sent out through e-mail). If it is an e-mail survey, were the responses entered manually into a database, or was there an automatic method for capturing responses?	Materials & Methods Page 1
	Context	Describe the Web site (for mailing list/newsgroup) in which the survey was posted. What is the Web	N/A

	Checklist for Reporting Results of Internet E-Surveys (CHERRIES)		
Item Category	***Checklist Item***	***Explanation***	
		site about, who is visiting it, what are visitors normally looking for? Discuss to what degree the content of the Web site could pre-select the sample or influence the results. For example, a survey about vaccination on a anti-immunization Web site will have different results from a Web survey conducted on a government Web site	
	Mandatory/voluntary	Was it a mandatory survey to be filled in by every visitor who wanted to enter the Web site, or was it a voluntary survey?	Materials & Methods Page 1
	Incentives	Were any incentives offered (eg, monetary, prizes, or non-monetary incentives such as an offer to provide the survey results)?	Materials & Methods Page 1
	Time/Date	In what timeframe were the data collected?	Materials & Methods Page 1
	Randomization of items or questionnaires	To prevent biases items can be randomized or alternated.	N/A
	Adaptive questioning	Use adaptive questioning (certain items, or only conditionally displayed based on responses to other items) to reduce number and complexity of the questions.	Materials & Methods Page 1-2
	Number of Items	What was the number of questionnaire items per page? The number of items is an important factor for the completion rate.	Materials & Methods Page 1
	Number of screens (pages)	Over how many pages was the questionnaire distributed? The number of items is an important factor for the completion rate.	N/A
	Completeness check	It is technically possible to do consistency or completeness checks before the questionnaire is submitted. Was this done, and if "yes", how (usually JAVAScript)? An alternative is to check for completeness after the questionnaire has been submitted (and highlight mandatory items). If this has been done, it should be reported. All items should provide a non-response option such as "not applicable" or "rather not say", and selection of one response option should be enforced.	N/A
	Review step	State whether respondents were able to review and change their answers (eg, through a Back button or a Review step which displays a summary of the responses and asks the respondents if they are correct).	N/A
**Response rates**			
	Unique site visitor	If you provide view rates or participation rates, you need to define how you determined a unique visitor. There are different techniques available, based on IP addresses or cookies or both.	Materials & Methods Page 1
	View rate (Ratio of unique survey visitors/unique site visitors)	Requires counting unique visitors to the first page of the survey, divided by the number of unique site visitors (not page views!). It is not unusual to have view rates of less than 0.1 % if the survey is voluntary.	N/A
	Participation rate (Ratio of unique visitors who agreed to	Count the unique number of people who filled in the first survey page (or agreed to participate, for example by checking a checkbox), divided by	Materials & Methods Page 1

	Checklist for Reporting Results of Internet E-Surveys (CHERRIES)		
Item Category	***Checklist Item***	***Explanation***	
	participate/unique first survey page visitors)	visitors who visit the first page of the survey (or the informed consents page, if present). This can also be called "recruitment" rate.	N/A
	Completion rate (Ratio of users who finished the survey/users who agreed to participate)	The number of people submitting the last questionnaire page, divided by the number of people who agreed to participate (or submitted the first survey page). This is only relevant if there is a separate "informed consent" page or if the survey goes over several pages. This is a measure for attrition. Note that "completion" can involve leaving questionnaire items blank. This is not a measure for how completely questionnaires were filled in. (If you need a measure for this, use the word "completeness rate".)	Results Page 1
**Preventing multiple entries from the same individual**			
	Cookies used	Indicate whether cookies were used to assign a unique user identifier to each client computer. If so, mention the page on which the cookie was set and read, and how long the cookie was valid. Were duplicate entries avoided by preventing users access to the survey twice; or were duplicate database entries having the same user ID eliminated before analysis? In the latter case, which entries were kept for analysis (eg, the first entry or the most recent)?	N/A
	IP check	Indicate whether the IP address of the client computer was used to identify potential duplicate entries from the same user. If so, mention the period of time for which no two entries from the same IP address were allowed (eg, 24 hours). Were duplicate entries avoided by preventing users with the same IP address access to the survey twice; or were duplicate database entries having the same IP address within a given period of time eliminated before analysis? If the latter, which entries were kept for analysis (eg, the first entry or the most recent)?	Materials & Methods Page 1
	Log file analysis	Indicate whether other techniques to analyze the log file for identification of multiple entries were used. If so, please describe.	Via Qualtrics during data analysis
	Registration	In "closed" (non-open) surveys, users need to login first and it is easier to prevent duplicate entries from the same user. Describe how this was done. For example, was the survey never displayed a second time once the user had filled it in, or was the username stored together with the survey results and later eliminated? If the latter, which entries were kept for analysis (eg, the first entry or the most recent)?	User was identified via IP address to ensure unique participants.
**Analysis**			
	Handling of incomplete questionnaires	Were only completed questionnaires analyzed? Were questionnaires which terminated early (where, for example, users did not go through all questionnaire pages) also analyzed?	Results Page 1. Incomplete questionnaires were not included.

	Checklist for Reporting Results of Internet E-Surveys (CHERRIES)	

Item Category	*Checklist Item*	*Explanation*	
	Questionnaires submitted with an atypical timestamp	Some investigators may measure the time people needed to fill in a questionnaire and exclude questionnaires that were submitted too soon. Specify the timeframe that was used as a cut-off point, and describe how this point was determined.	N/A
	Statistical correction	Indicate whether any methods such as weighting of items or propensity scores have been used to adjust for the non-representative sample; if so, please describe the methods.	N/A
